# Carfilzomib preserves dendritic cell phenotype and function compared with bortezomib

**DOI:** 10.3389/fimmu.2026.1936021

**Published:** 2026-09-22

**Authors:** L. Wang, M. Dahlfrancis, D. Dörfel, J. S. Walz, M. G. Klatt, F. Grünebach, S. M. Rittig

**Affiliations:** 1 Department of Hematology, Oncology and Cancer Immunology, Charité - Universitaetsmedizin Berlin, Corporate Member of Freie Universitaet Berlin and Humboldt-Universitaet zu Berlin, Berlin, Germany; 2 Department for Hematology, Oncology, Clinical Immunology and Rheumatology, University Hospital Tuebingen, Tuebingen, Germany; 3 Department of Otolaryngology, Head and Neck Surgery, University Hospital Essen, Essen, Germany; 4 Department of Medical Oncology, Hematology and Immunology, KRH Hospital Siloah, Hannover, Germany; 5 Clinical Collaboration Unit Translational Immunology, Department of Internal Medicine, University Hospital Tuebingen, Tuebingen, Germany; 6 German Cancer Consortium (DKTK) and German Cancer Research Center (DKFZ), Partner Site Tuebingen, Tuebingen, Germany; 7 Department of Peptide-based Immunotherapy, Institute of Immunology, University and University Hospital Tuebingen, Tuebingen, Germany; 8 Cluster of Excellence iFIT (EXC2180) “Image-Guided and Functionally Instructed Tumor Therapies”, University of Tuebingen, Tuebingen, Germany; 9 BIH Charité Clinician Scientist Program, Berlin Institute of Health (BIH) at Charité - Universitaetsmedizin Berlin, Berlin, Germany

**Keywords:** cancer vaccines, carfilzomib, dendritic cells, immune checkpoint inhibition, immunogenic cell death, immunotherapy, multiple myeloma, proteasome inhibitors

## Abstract

**Introduction:**

Despite major therapeutic advances, multiple myeloma (MM) remains largely incurable. Moreover, side effects and toxicities of treatments compromise patients’ quality of life. Dendritic cell (DC)-based cancer vaccines can induce long-term tumor-specific immune responses and are generally well tolerated. To improve the potency of this immunotherapeutic approach, researchers are now exploring combination strategies that integrate approved agents with DC-based vaccines. Proteasome inhibitors (PIs), including bortezomib and carfilzomib, are the standard of care for MM. PIs have been shown to induce so-called immunogenic cell death and could therefore be promising agents for combination approaches. However, careful selection of the optimal drug is mandatory, as off-target effects may also hamper the immune system, as previously shown for bortezomib.

**Methods:**

By flow cytometry, we systematically evaluated the effects of the PI carfilzomib on the phenotype and expression of immune checkpoint molecules in monocyte-derived human DCs (moDCs) compared to bortezomib. Functional analyses included endocytosis, migration, cytokine secretion, and T cell stimulatory capacity in mixed lymphocyte reactions (MLRs), with and without PD-1 blockade. We assessed DC frequencies in peripheral blood and bone marrow of MM patients at diagnosis, during carfilzomib-based therapy, and in remission and further generated DCs using monocytes of carfilzomib-treated patients.

**Results:**

Carfilzomib exposure did not compromise moDC characteristics, including phenotype, endocytosis, migration, and cytokine secretion, in vitro, whereas bortezomib-exposed moDCs differed significantly in several parameters. Carfilzomib modestly increased PD-L1 expression on moDCs and significantly reduced both their allostimulatory capacity and the IFN-γ production of cocultured T cells. PD-1 blockade with nivolumab fully reversed this effect and significantly enhanced it in MLR. We assessed reduced numbers of circulating DCs in MM patients at diagnosis, but numbers were comparable to those in healthy donors, patients receiving carfilzomib-based therapy and patients in remission. Furthermore, we could generate moDCs with a typical surface phenotype from monocytes of patients receiving carfilzomib-based therapy.

**Conclusion:**

Our findings show that carfilzomib does not cause the broad impairment seen with bortezomib, and that the discrete deficit it does produce is reversible with PD-1 blockade. We hypothesize that carfilzomib is a more suitable proteasome inhibitor partner for DC-based immunotherapeutic strategies.

## Introduction

MM accounts for about 10% of all hematological malignancies and is characterized by unregulated clonal plasma cell growth within the bone marrow, leading to profound immune dysregulation (1). Approval of a variety of therapeutics with meaningful efficacy, such as proteasome inhibitors, immunomodulatory drugs, monoclonal antibodies, bispecific antibodies, antibody-drug conjugates, and chimeric antigen receptor (CAR)-T cell therapy, has substantially improved outcomes (2). However, long-term remissions are very few, with 5-year survival rates around 62%. Relapse and the development of drug resistance continue to pose major clinical challenges (3, 4), and cumulative toxicity limits the feasibility of continuous treatment, particularly in elderly or frail patients. These challenges highlight the need for therapeutic strategies that induce durable disease control while preserving quality of life.

Therapeutic cancer vaccines aim to induce long-lasting, tumor-specific immune responses and generally have favorable safety profiles.

DCs are recognized as the most potent specialized antigen-presenting cells. They are central to this immunotherapeutic approach due to their unique capacity to prime naïve T cells and to both initiate and regulate antigen-specific immune responses (5).

In their immature state, DCs scan the body for infected or transformed cells, detect and capture pathogens and antigens, and present them to T cells. With the help of pattern recognition and endocytosis receptors, they internalize antigens via endocytosis (5), which are then processed. Upon activation, DCs upregulate the C-C chemokine receptor type 7 (CCR7), which guides their migration to secondary lymphoid organs such as lymph nodes. Upon arrival, they present processed antigens via major histocompatibility complex (MHC) molecules to naïve CD8+ and CD4+ T cells. DCs, then referred to as mature DCs, express high levels of MHC complexes and costimulatory molecules and secrete cytokines and chemokines, including interleukin 12 (IL-12), IL-6, C-C motif chemokine ligand (CCL) 2, 3 and 5, which are essential for the activation and differentiation of effector T cells (5, 6).

Several DC subtypes are classified based on their development, distinct phenotypes, and functional roles in immunity (7). Conventional DCs (cDCs) comprise cDC1 (CD141+) and cDC2 (CD1c+) subsets (8). In anti-cancer immunity, cDC1 play an important role due to their high efficiency in cross-presenting tumor antigens to CD8+ T cells. cDC2, on the other hand, have been shown to potently induce helper CD4+ T cell-mediated immune responses (7), while plasmacytoid DCs (pDCs), characterized by CD123 and CD303 expression, are primarily involved in antiviral responses and are major producers of type I interferons (8).

Given their central role in priming and regulating immune responses, DCs are key players in immunotherapeutic strategies, particularly but not only in therapeutic cancer vaccines. Cancer vaccines can be based on peptides, proteins, DNA, or mRNA encoding tumor antigens (6). Monocyte-derived DCs (moDCs) are commonly used, as they are easy to generate in sufficient numbers *in vitro* and can be loaded with tumor antigens. Alternatively, tumor antigen-encoding mRNA or peptides derived from tumor-associated antigens (TAAs) can be administered directly. These approaches rely on DCs to capture the antigens *in vivo* and induce tumor-specific T cell responses (9).

DC-based vaccines have demonstrated immunogenicity in MM and other malignancies in the past decades (5). However, exception for sipuleucel-T in metastatic castrate-resistant prostate cancer, clinical efficacy has remained modest (10–13). In MM, a phase I trial by Rosenblatt et al. demonstrated disease stabilization in patients vaccinated with autologous moDCs, fused with the patient’s myeloma cells. However, long-term survival could not be achieved (14). Similarly, a phase II trial employing DCs loaded with idiotype protein failed to achieve clinical efficacy (15). The limited success in MM has been attributed to advanced disease stage at vaccination, tumor-induced immunosuppression, and functional impairment of DCs and T cells within the myeloma microenvironment (16, 17).

Consequently, current efforts increasingly focus on combinatorial strategies that integrate DC-based vaccination with immune-modulating agents capable of reshaping the tumor microenvironment. Proteasome inhibitors (PIs) represent an attractive component of such combinations, as MM cells are highly dependent on proteasomal protein degradation. By inhibiting the proteasome, polyubiquitinated proteins accumulate, leading to induction of apoptosis, cell cycle arrest, and inhibition of tumor proliferation (18). Hence, PIs such as bortezomib and carfilzomib are a cornerstone of first-line therapy.

Although both bortezomib and carfilzomib comparably inhibit the chimeric proteasome-like activity, carfilzomib shows greater specificity for the N-terminal catalytic threonine residues of the proteasome, thereby reducing off-target effects (18). In addition, unlike bortezomib, a selective and reversible proteasome inhibitor, its irreversible binding allows carfilzomib to achieve long-term efficacy and maintain clinical response in cases of bortezomib resistance (19). Moreover, carfilzomib is thought to induce so-called immunogenic cell death (ICD) characterized by the release of TAA and damage-associated molecular patterns that promote DC recruitment, maturation and antigen presentation (20), eventually leading to the induction of an adaptive anti-tumor immune response (21, 22). Given this potential, carfilzomib may serve as an ideal agent for combination approaches. However, its irreversible binding to the proteasome could contribute to off-target immunosuppressive effects, particularly on DCs, as previously shown for bortezomib (23).

Combining DC vaccines with immune checkpoint inhibitors (ICIs) represents a further promising strategy to overcome T cell exhaustion and augment anti-tumor responses (14, 24, 25). Approved ICIs targeting programmed cell death protein 1 (PD-1), programmed cell death ligand 1 (PD-L1), and cytotoxic T-lymphocyte-associated antigen 4 (CTLA-4) have demonstrated durable responses across a wide range of cancers (25, 26). These agents block inhibitory checkpoint interactions between tumor cells or antigen-presenting cells (including DCs) and T cells, thereby releasing the brake and restoring T cell activation (27). However, a phase I clinical trial evaluating the PD-1 inhibitor nivolumab as monotherapy in MM failed to achieve convincing clinical efficacy, potentially due to the immunosuppressive tumor microenvironment in MM (28). Several ongoing clinical trials are exploring combinatorial strategies, including the integration of cancer vaccines with ICIs and other immune-modulating agents. E.g., a Phase II clinical trial (NCT01067287) is investigating the combination of a DC fusion vaccine and CT-011 (an investigational anti-PD-1 monoclonal antibody) after autologous stem cell transplantation in MM patients (17).

Here, we systematically investigated the effects of carfilzomib on human moDC phenotype, immune checkpoint expression, and functional capacity, in direct comparison to bortezomib. We further examined whether immune checkpoint inhibition could enhance moDC-mediated T cell activation in the context of carfilzomib exposure. Finally, we assessed the feasibility of generating moDCs from MM patients receiving carfilzomib as part of their therapeutic regimen, and analyzed the impact of disease and treatment on circulating DC subsets. We aimed to establish a rational basis for a combination approach using a DC-based vaccine, carfilzomib and ICIs to achieve durable, effective, and well-tolerated responses in MM patients.

## Materials and methods

### Generation of moDCs

Generation of moDCs from adherent monocytes was carried out as previously described (29, 30). In brief, peripheral blood mononuclear cells (PBMCs) were isolated from buffy coats or peripheral blood of healthy donors (obtained from the blood bank of Tuebingen and Charité) or from patients undergoing carfilzomib treatment by Ficoll (Thermo Fisher Scientific, Waltham, USA) density gradient centrifugation after informed consent. The local ethics committees (Charité: EA4/237/21 and University of Tuebingen: 344/2008BO2) approved all procedures. PBMCs were resuspended in X-VIVO (Lonza, Basel, Switzerland) and allowed to adhere in 25 cm^2^ cell culture flasks (BD Falcon, Heidelberg, Germany) at 37 °C and 5% CO_2_ at a concentration of 0.8-1*10^7^ cells/ml. After 2 h, non-adherent cells were rinsed off and adherent monocytes were cultured in RPMI 1640 medium (supplemented with 10% heat-inactivated fetal calf serum (FCS) and 2% penicillin/streptomycin). Granulocyte-macrophage colony-stimulating factor (GM-CSF) (100 ng/ml; ImmunoTools, Friesoythe, Germany) and IL-4 (20 ng/ml; PeproTech, Cranbury, USA) were added every other day for six days to generate immature moDCs. Lipopolysaccharide (LPS) (TLR4L, 100 ng/ml; Sigma-Aldrich, Deisenhofen, Germany) was added for the final 16 hours to induce maturation (31). Cells were harvested with cold PBS on day 7, yielding mature moDCs (LPS-treated) or immature moDCs (without LPS) for further analysis.

Bortezomib (1 ng/ml; Selleckchem, Houston, USA), dissolved in DMSO, was added to the cell culture 24 h before harvest as previously published on day 6 (23). Carfilzomib (100 nM, Selleckchem, Houston, USA), dissolved in DMSO, was added to the medium for 60 min on day 6. The doses were administered using fresh cell culture medium supplemented with IL-4 and GM-CSF. Specifically, cells were separated from medium via centrifugation, which was preserved for later resuspension of the cells back into their original microenvironment. The carfilzomib-containing medium was added to the flask, and the cells were incubated for 60 minutes. Following incubation, the medium was then separated from the cells by centrifugation and the flasks were washed twice with RPMI medium. The small number of non-adherent cells was washed twice with RPMI and added to the adherent DCs in the flask, followed by the addition of the preserved medium.

Drug concentrations and exposure times were chosen to resemble clinical application and were based on pharmacokinetic data from previous studies and IC_50_ analyses (32–34). Vehicle-treated cells served as negative controls, while IL-10 (10 ng/ml; R&D Systems) added every other day starting on day 0 served as the positive control.

### Cell viability

Apoptosis of moDCs and U266 multiple myeloma cells (obtained from the Leibniz Institute DSMZ - German Collection of Microorganisms and Cell Cultures GmbH, Braunschweig, Germany) under exposure to carfilzomib was assessed by Annexin V/propidium iodide staining and flow cytometry (Staining Kit, Roche Diagnostics GmbH, Mannheim, Germany). DMSO and staurosporine (500 nM; R&D Systems, Wiesbaden, Germany) served as negative and positive controls, respectively.

### Proteasome activity assays

To assess potential effects of β5 proteasome inhibition on DC maturation and differentiation immature and mature moDCs pretreated with 100 nM carfilzomib (1 h), 1 ng/mL bortezomib (24 h), or DMSO control on day 6 were harvested on day 7 (24 h after beginning of PI exposure and 16 h after addition of LPS in mature moDC), washed, and lysed by one cycle of freezing and thawing. 50 μg protein was used per reaction. The activity of the proteasome’s β5 subunit was measured using Proteasome-Glo Chymotrypsin-like assay (Promega, G8621) according to the manufacturer’s instructions. The luminescence signal was detected after incubation with the Proteasome-Glo reagent. β5 activity was normalized to the DMSO control. Assays were performed in three technical replicates for each of three independent healthy donors.

### Flow cytometry

Surface marker expression of moDCs was analyzed using fluorochrome-conjugated monoclonal mouse anti-human antibodies including FITC-, PE-, PerCP-Cy5.5- and APC against CD1a, CD14, CD209, CD86, PD-L1, PD-L2, Galectin-3 (Gal-3), HLA-ABC (BD Biosciences), CD80, CD83, HLA-DR, Osteoactivin (GPNMB; all eBioscience), CCR7 (R&D Systems), and T cell immunoglobulin and mucin domain containing 3 (TIM-3) (BioLegend) along with corresponding mouse IgG isotype controls (BD Biosciences; R&D Systems). Data were acquired on a FACSCalibur cytometer (Beckman Coulter) and analyzed using FlowJo software. The delta of median fluorescence intensity (DMFI) between isotype control and a specific molecule served as a stand-in value for the expression of the analyzed surface molecules. To enhance comparability across experiments, normalized DMFI was calculated by dividing the DMFI of the treated cells by that of the DMSO control cells.

### Functional assays

Endocytic capacity was assessed by FITC-dextran uptake in immature moDCs. 1*10^5^ moDCs were harvested and incubated with 5 µl FITC-dextran (Sigma-Aldrich, Deisenhofen, Germany) at 37 °C and 5% CO_2_. After one hour the cells were washed, and FITC-dextran uptake was measured by fluorescence-activated cell sorter (FACS) analysis.

Migratory capacity was analyzed using transwell assays toward CCL19. For this, mature moDCs were suspended at a concentration of 2*10^5^ per ml in serum-free RPMI cell culture medium and transferred into transwell chambers (8 μm polycarbonate membrane pore; Corning) placed in 24-well plates (BD Falcon, Heidelberg, Germany). The bottom well of each plate was filled with 1 ml of RPMI serum-free medium containing CCL19/MIP-3β (100 ng/ml; R&D Systems). Cells were allowed to migrate for 16 hours at 37 °C and 5% CO_2_, and the number of migrated cells was analyzed by FACS in a previously established gate for 1 minute.

Supernatants from pretreated mature moDC cultures were collected on day seven and stored at -80 °C for further analysis. Cytokine levels of CCL-2, CCL-3, CCL-5, IL-6, and IL-12 were measured using enzyme-linked immunosorbent assay (ELISA) kits (R&D Systems; BD Biosciences), according to the manufacturer’s instructions.

### Mixed lymphocyte reaction

Allogenic human T cells were isolated from healthy donors and cultured in RPMI 1640 medium supplemented with 10% heat-inactivated FCS and 1% penicillin/streptomycin. The T cells were pre-activated using CD3/CD28 Dynabeads (Thermo Fisher Scientific, 11-161-D) for 24 hours. Pre-activation was necessary to obtain reproducible proliferation within a five-day co-culture given the low frequency of alloreactive precursors and the limited moDCs available. After three days of culture, the pre-activated T cells were stained with carboxyfluorescein succinimidyl ester (CFSE) (BD Biosciences). The PD-1 inhibitor nivolumab (10 μg/ml; MedChemExpress; Bristol Myers Squibb) or a human IgG4 isotype control was added to the T cells. Mature moDCs were inactivated by 30 Gy irradiation and co-cultured with T cells at a ratio of 1:10 in a 96-well plate with 2*10^4^ moDCs per well. T cell proliferation was assessed by flow cytometry after five days.

To further assess T cell function, supernatants were collected on day 5 after co-culture. Interferon-gamma (IFN-γ) concentrations were measured using a human IFN-γ ELISA kit (R&D Systems), according to the manufacturer’s instructions.

### Patient samples

Peripheral blood and bone marrow samples were obtained at Charité - Universitaetsmedizin Berlin between September 2022 and December 2023 from newly diagnosed MM patients, patients receiving carfilzomib-based therapy, and patients in remission. All participants provided written informed consent, and the analyses were approved by institutional ethics boards in accordance with the latest Helsinki Declaration. For newly diagnosed MM patients, the following laboratory tests and imaging examinations were performed by the local laboratory before the initiation of anti-myeloma treatment: serum monoclonal protein, free light chains, calcium, creatinine, lactate dehydrogenase (LDH) levels, type of M-protein, percentage of clonal bone marrow plasma cells, scan for osteolytic bone lesions, chromosome analysis, hemoglobin and white blood cell counts. Patients were staged according to the revised international staging system (R-ISS). Additionally, peripheral blood and bone marrow samples were collected from newly diagnosed patients before they began any anti-tumor treatment. For patients receiving carfilzomib, we obtained peripheral blood samples after 4 cycles of first-line treatment.

### Cell frequencies of blood DC subtypes

DC subsets in peripheral blood and bone marrow were identified by flow cytometry based on established marker combinations. Red blood cells were lysed by ACK lysing buffer (Thermo Fisher Scientific, Waltham, USA) for 5–10 minutes. White blood cells (WBCs) were stained with the following antibodies: CD45, CD3, CD19, CD14, CD16, HLA-DR, CD11c, CD1c, CD141, CD123 and CD303. CD138 antibody was added for the staining of plasma and myeloma cells in bone marrow analysis. Data analysis was performed using FlowJo software (**Supplementary Figure 3**).

### Statistical analysis

Data are presented as mean ± standard deviation (SD). Outliers were identified using the ROUT method in GraphPad Prism and excluded from the final analysis. The relative differences among three or more groups for a single variable were analyzed using one-way ANOVA with treatment conditions and the DMSO control. The Mann-Whitney U test was applied to nonparametric data across two groups. Clinical data analyses were performed using SPSS software (version 20.0; SPSS Inc, Chicago, IL, USA). For analyses of MM parameters and total DC numbers in PBMCs, continuous variables were compared using the Spearman correlation test. Binary variables were analyzed using the Mann-Whitney U test. Continuous variables across multiple groups were compared using one-way or two-way analysis of variance (ANOVA) where appropriate. Comparisons for which no statistically significant difference was detected are reported as such and should not be interpreted as showing equivalence. No formal equivalence or non-inferiority testing was performed. A p-value < 0.05 was considered statistically significant.

## Results

### Dose finding

Apoptosis rates in U266 MM cell line exceeded 80% at carfilzomib concentrations of 100 nM or higher (**Supplementary Figures 1A, C**). In contrast, both immature and mature moDCs showed apoptosis rates of below 10% at 100 nM concentration. Based on our own data and previous studies reporting IC_50_ values for multiple myeloma cell lines and pharmacokinetic data in patients, we selected 100 nM as the experimental concentration (**Supplementary Figures 1B, C**) (32–34). To mimic *in vivo* conditions, moDCs were exposed to proteasome inhibitors only once during culture.

### Carfilzomib and bortezomib inhibit β5 proteasome activity in moDCs

To assess proteasome target engagement, we measured β5 proteasome activity in moDCs under carfilzomib and bortezomib treatment. Carfilzomib markedly inhibited β5 activity by 64.6% in immature and 66.7% in mature moDCs, and bortezomib by 60.6% and 62.3% respectively. Notably, the β5 inhibition achieved by a 1 h exposure of moDCs to carfilzomib was at least as strong as that induced by a 24 h exposure to bortezomib in every donor and every replicate. The differences in moDC phenotype and function observed here are hence not due to weaker proteasome target engagement by carfilzomib (**Supplementary Figure 2**).

### Surface marker expression of moDCs is largely unchanged after carfilzomib exposure

Given its irreversible inhibition of the ubiquitin-proteasome pathway, carfilzomib may exert off-target effects on immune cells, as previously shown for the PI bortezomib (23). To assess potential effects of β5 proteasome inhibition on DC maturation and differentiation, moDCs were treated with 100 nM carfilzomib or 1 ng/ml bortezomib, respectively, on day 6 of culture. Phenotypic analyses of mature and immature moDCs were performed on day 7 by flow cytometry.

Neither PI affected total moDC yield (data not shown). Compared to DMSO-treated controls, moDCs exposed to carfilzomib or bortezomib showed only minor morphological changes as assessed by light microscopy and flow cytometry (cell size and granularity). In contrast, IL-10 (positive control) markedly altered the morphology of both mature and immature moDCs, resulting in reduced cell size, decreased intracellular granularity (**Figure 1A**), and restricted dendrite formation (data not shown).

**Figure 1:**
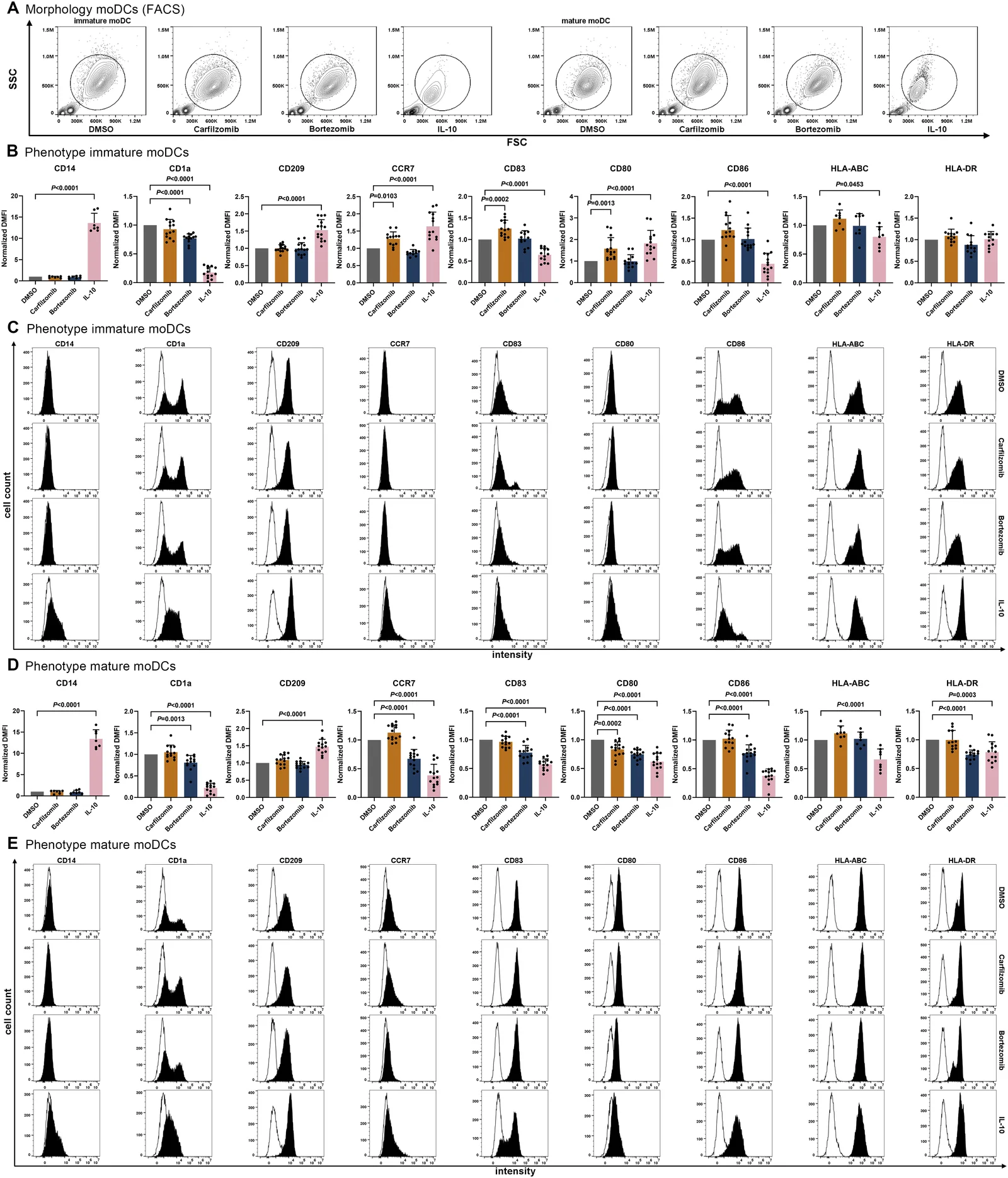
Effects of carfilzomib and bortezomib on morphology and phenotype of moDCs. MoDCs were generated *in vitro* for 7 days with or without LPS stimulation. On day 6, cells were treated with DMSO or bortezomib (1 ng/ml) for 24 hours, or with carfilzomib (100 nM) for 1 hour. On day 7, moDC size and granularity were analyzed by FACS. **(A)** Exemplarily, one representative of 15 replicable experiments is depicted. Expression of the indicated cell surface markers characterizing moDC phenotype was further assessed by FACS. **(B–E)** Levels of expression are shown as normalized DMFI. Statistical analysis (B: immature moDCs and D: mature moDCs) and exemplary data (C: immature moDCs and E: mature moDCs) are shown. Matched isotype controls are presented as histograms. The numbers of biological replicates were as follows: CD14 (n = 7), CD1a (n = 13), CD209 (n = 14), CCR7 (n = 14), CD83 (n = 14), CD80 (n = 15), CD86 (n = 14), HLA-ABC (n = 8), HLA-DR (n = 13). (One-way ANOVA, Mean ± SD).

To further evaluate potential effects of proteasome inhibition on DC maturation and differentiation, we analyzed surface markers. Statistical analyses of surface marker expression are presented in **Figures 1B, D** (n = 7-15). Representative flow cytometry plots for immature and mature moDCs are displayed in **Figures 1C, E**, respectively. During differentiation, the monocyte marker CD14 was downregulated in all experimental conditions except IL-10, indicating significantly impaired DC differentiation in moDCs exposed to IL-10 (**Figures 1B–D**).

Following differentiation, untreated moDCs exhibited a typical phenotype characterized by high CD1a expression, a surface protein involved in the presentation of self- and pathogen-derived lipid antigens to T cells. DCs highly expressing CD1a are known to produce substantial amounts of the T cell stimulatory cytokine IL-12 (35, 36). CD1a expression was comparable to controls in both immature and mature moDCs exposed to carfilzomib, but was significantly reduced following bortezomib treatment, and was very low in IL-10-treated cells.

Expression of CD209 (DC-SIGN: DC-specific ICAM-3-grabbing nonintegrin), a C-type lectin involved in recognition and internalization of antigens, but also in the interaction with T cells and in shaping of the immune response (37), was not affected by either PI.

Interestingly, the chemokine receptor CCR7, which mediates DC migration toward lymph nodes in response to CCL19 (38), was significantly upregulated in immature moDCs exposed to carfilzomib, while bortezomib led to reduced expression of CCR7 in mature moDCs.

The maturation marker CD83 was significantly increased in immature moDCs following carfilzomib treatment, but was significantly decreased by bortezomib exposure in mature moDCs (39). Expression of the costimulatory molecules CD80 and CD86 was markedly reduced after bortezomib treatment in LPS-stimulated moDCs, whereas carfilzomib induced only a minor, though statistically significant, reduction of CD80 levels. Finally, we evaluated the expression of MHC class I (HLA-ABC) and MHC class II (HLA-DR) molecules, which are essential for antigen presentation and T cell activation. Neither PIs significantly affected HLA-ABC expression; however, bortezomib led to a statistically significant down-regulation of HLA-DR in mature moDCs (**Figures 1B–E**).

In summary, in contrast to bortezomib, exposure to carfilzomib does not substantially impair surface marker expression or maturation of moDCs, with the exception of a slight but less pronounced reduction of CD80 expression in mature moDCs compared to bortezomib.

### Effect of carfilzomib exposure on checkpoint molecule expression in moDCs

DCs regulate the immune response not only through costimulatory signals but also via immune checkpoint molecules (40). We hypothesized that modulation of checkpoint inhibitor expression by PIs may influence the T cell stimulatory capacity of DCs and hence analyzed the surface expression of selected immune checkpoint molecules on moDCs exposed to PIs.

Exposure to both carfilzomib and bortezomib led to a statistically significant increase in PD-L1 surface expression in mature as well as immature DCs. In addition, carfilzomib treatment led to modestly upregulated PD-L2 expression in unstimulated moDCs. TIM-3, a further immune checkpoint, was shown to limit cDC1 function by suppressing extracellular DNA sensing and antigen uptake, thereby negatively regulating T-helper cell responses (41, 42). TIM-3 levels were significantly reduced in immature moDCs following carfilzomib treatment, whereas IL-10 exposure led to increased TIM-3 levels. Osteoactivin (GPNMB) also functions as an immune checkpoint acting as a negative regulator of T cell activation. Our group previously demonstrated that upregulation of GPNMB under imatinib treatment significantly impaired DC stimulatory capability in MLR, an effect that could be reversed and even significantly enhanced by GPNMB blockade (43). Gal-3, the ligand of lymphocyte-activation gene 3 (LAG-3), contributes to immunosuppression by impairing the function of tumor-specific cytotoxic T lymphocytes by distancing the TCR from CD8 (44). Expression of GPNMB and Gal-3 was not significantly altered by proteasome inhibitor treatment, while GPNMB (as previously shown) was profoundly increased following IL-10 exposure (**Figures 2A–D**, n = 9-15). In conclusion, exposure to either PI leads to increased PD-L1 expression on moDCs, while carfilzomib additionally reduces TIM-3 expression in immature moDCs.

**Figure 2:**
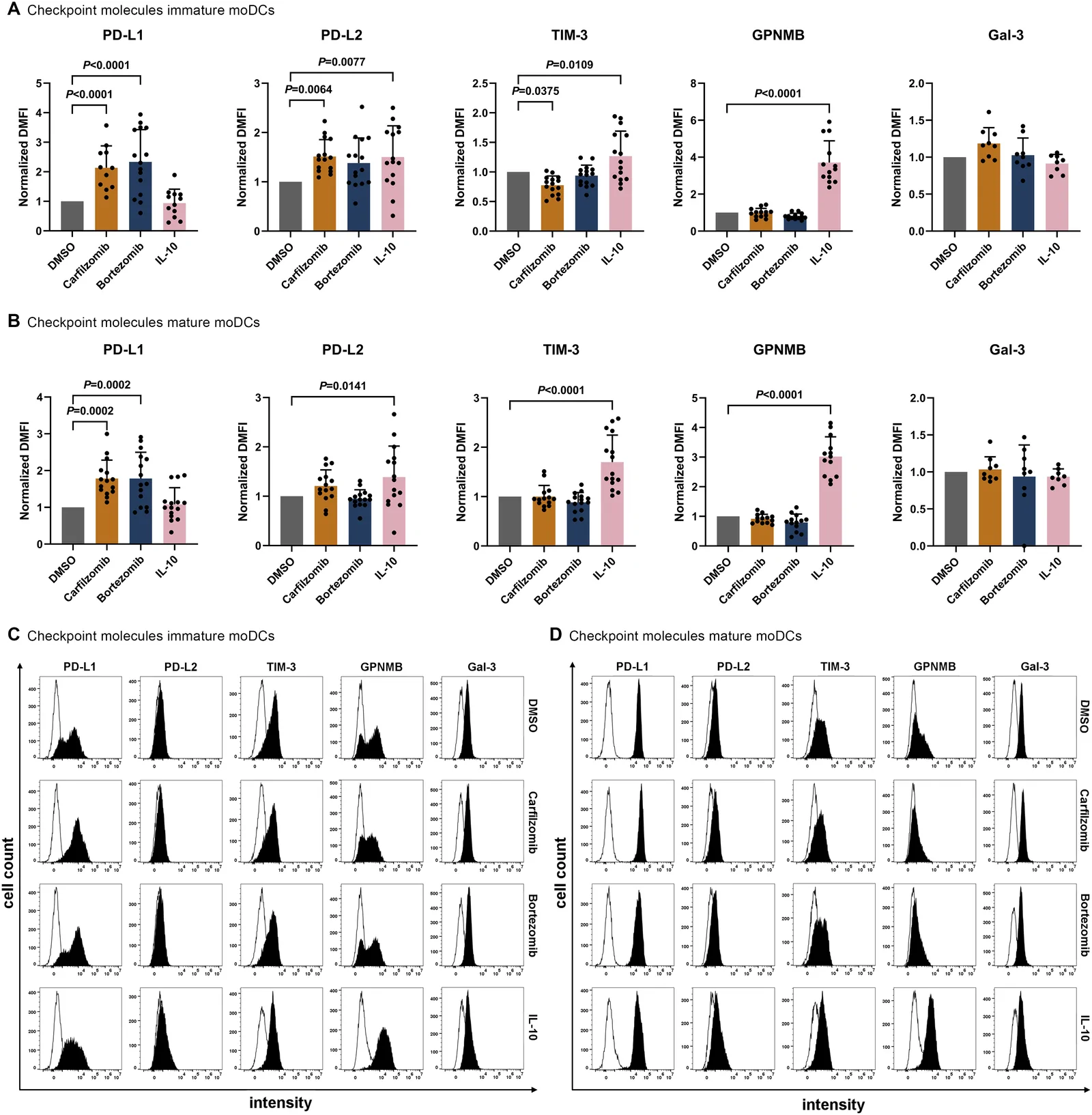
Effects of carfilzomib and bortezomib on immune checkpoint molecule expression in moDCs. Expression of the indicated immune checkpoint molecules on immature or mature moDCs was assessed after exposure to carfilzomib and bortezomib, and compared with DMSO and IL-10 controls by FACS. Expression levels are shown as normalized DMFI. Statistical analysis [**(A)** immature moDCs and **(B)** mature moDCs] and exemplary data [**(C)** immature moDCs and **(D)** mature moDCs] are shown. The numbers of biological replicates were as follows: PD-L1 (n = 15), PD-L2 (n = 15), TIM-3 (n = 15), GPNMB (n = 13), Gal-3 (n = 9). (One-way ANOVA, Mean ± SD).

### Endocytic capacity is not significantly altered by exposure to either PI

DCs patrol the body and are specialized in recognizing foreign as well as tumor antigens. In their immature state, DCs take up antigens via endocytosis, which is followed by their processing to peptides and presentation as MHC-peptide complexes, ultimately leading to the initiation of specific T cell responses. To evaluate a possible influence of the PIs on the key function of endocytic capacity, we analyzed the internalization of FITC-labeled dextran in pretreated immature DCs (45). We did not observe statistically significant influence on endocytosis under carfilzomib or bortezomib exposure compared to the DMSO control (**Figure 3A**, n = 6).

**Figure 3:**
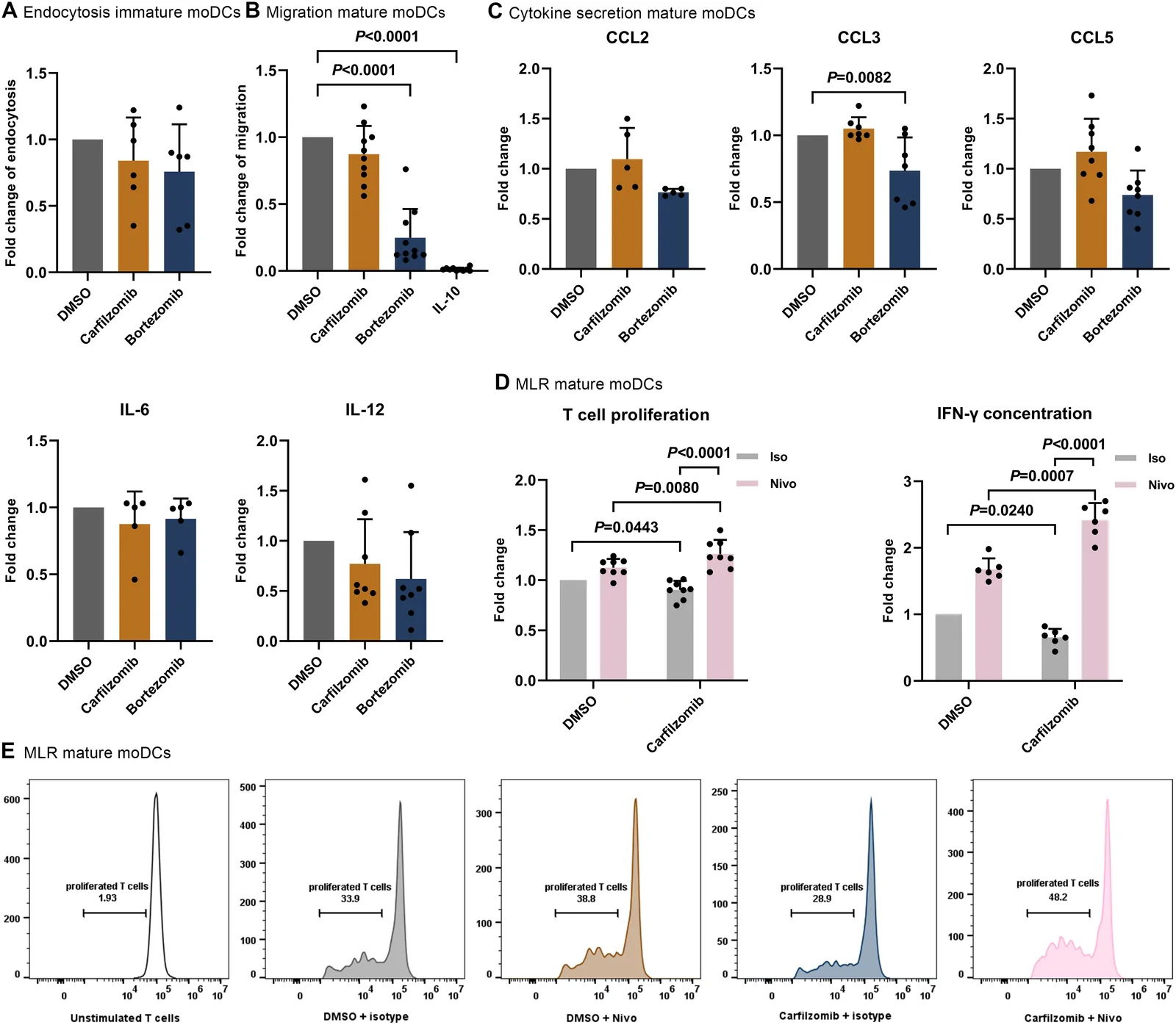
Effects of carfilzomib and bortezomib on moDC function. **(A)** Endocytic activity of immature moDCs treated with carfilzomib or bortezomib, determined by one-hour FITC-dextran uptake via flow cytometry. Pooled data from six independent biological replicates (n = 6) are shown. (One-way ANOVA, Mean ± SD, no statistical significance). **(B)** Migration of mature moDCs under treatment with carfilzomib or bortezomib and LPS stimulation, assessed by transwell assay to CCL19 using flow cytometry. Pooled results consisting of ten independent experiments (n = 10) are shown. (One-way ANOVA, Mean ± SD). **(C)** Cytokine release of mature moDCs treated with bortezomib and carfilzomib. Detection of CCL2, 3, 5 and IL-6, 12 released by moDCs was performed using ELISA, measuring absolute cytokine concentrations in cell culture supernatants. The numbers of biological replicates were as follows: CCL2 (n = 5), CCL3 (n = 7), CCL5 (n = 8), IL-6 (n = 5), IL-12 (n = 8). (One-way ANOVA, Mean ± SD). **(D, E)** T cell proliferation and IFN-γ production of mature carfilzomib-treated moDCs in the presence of an anti-PD-1 antibody. MLR of moDCs pretreated with carfilzomib or DMSO control with allogenic T cells was performed in the presence or absence of an ICI, anti-PD-1 antibody (nivolumab), with human IgG4 serving as the isotype control. T cell proliferation was assessed by FACS after five days of co-culture, and IFN-γ concentrations in the culture supernatants were measured by ELISA. Representative results **(D)** from eight independent experiments (n = 8) for T-cell proliferation and from six independent experiments (n = 6) for IFN-γ production are shown, along with exemplary data **(E)**. (Two-way ANOVA, Mean ± SD).

### Migratory capacity of moDCs is unchanged by carfilzomib exposure

Following antigen capture, the maturation process is initiated, and DCs acquire the capability to migrate to the draining lymph node, thereby upregulating the surface marker CCR7, which directs migration toward CCL19/MIP-3β gradients. In line with previously published data, we observed a significantly impaired migratory capacity of LPS-stimulated mature moDCs exposed to bortezomib, as demonstrated in transwell migration assays (**Figure 3B**, n = 10, P < 0.0001) (23). In contrast, migration of mature moDCs exposed to carfilzomib was not significantly altered.

### Carfilzomib exposure does not lead to suppressed moDC cytokine secretion

After antigen stimulation and during maturation, DCs secrete several cytokines that recruit and polarize additional immune cells to coordinate the immune response. To assess this function, we measured levels of MCP-1/CCL2, MIP-1α/CCL3, Rantes/CCL5, IL-6, and IL-12 in the culture supernatants of mature moDCs using ELISA. Exposure to bortezomib resulted in reduced levels of CCL3 (P = 0.0082), with no significant changes observed in CCL2, CCL5, IL-6, and IL-12 (**Figure 3C**). In contrast, carfilzomib treatment did not significantly alter cytokine secretion compared to the negative control (**Figure 3C**, n = 5-8).

### Carfilzomib slightly reduces moDC-mediated T cell stimulation, which is reversed and even enhanced by nivolumab

Being the most relevant DC function, we analyzed the capability of moDCs to induce allogenic T cell responses. Bortezomib was previously shown to hamper allostimulatory capacity in mature moDCs (23). Mature moDCs pretreated with carfilzomib or DMSO were co-cultured with allogenic T cells in the presence of nivolumab or IgG4 control, generating a paired 2 × 2 experimental design. T cell proliferation analysis revealed a modest but statistically significant reduction in the immunostimulatory capacity of moDCs exposed to carfilzomib alone (P = 0.0443). Notably, addition of nivolumab led to significantly enhanced (approximately 1.4-fold) T cell proliferation of carfilzomib-pretreated moDCs (P < 0.0001). This was even higher than nivolumab treatment alone (P = 0.0080). Two-way ANOVA revealed a significant interaction between carfilzomib and nivolumab (P = 0.0007; **Figures 3D, E**).

We further assessed IFN-γ concentration in cell culture supernatant as a functional response of T cell stimulation. Consistent with the T cell proliferation data, carfilzomib treatment alone was associated with significantly lower IFN-γ production (P = 0.0240). Here too, addition of nivolumab elicited a markedly increased (approximately 3.6-fold) IFN-γ production in carfilzomib-pretreated moDCs (P<0.0001), significantly higher than in the nivolumab-only group (P = 0.0007). A significant carfilzomib × nivolumab interaction was also observed in IFN-γ production (P = 0.0001; **Figure 3D**).

### MoDCs with a typical surface phenotype can be generated from monocytes of carfilzomib-treated patients

A prerequisite for DC-based cancer vaccines is the generation of sufficient numbers of functional autologous moDCs. Hence, as a next translational step, we generated moDCs from PBMCs of three MM patients who had received more than 4 cycles of carfilzomib/lenalidomide/dexamethasone (KRd) as their first-line treatment. DAPI staining revealed no significant influence of carfilzomib-containing regimens on moDC viability or moDC yield (data not shown). Furthermore, cell size and granularity, assessed by forward scatter (FSC)/side scatter (SSC) gating (**Figure 4A**), as well as the expression of 14 analyzed surface markers, were comparable to those of healthy controls (**Figure 4B**).

**Figure 4:**
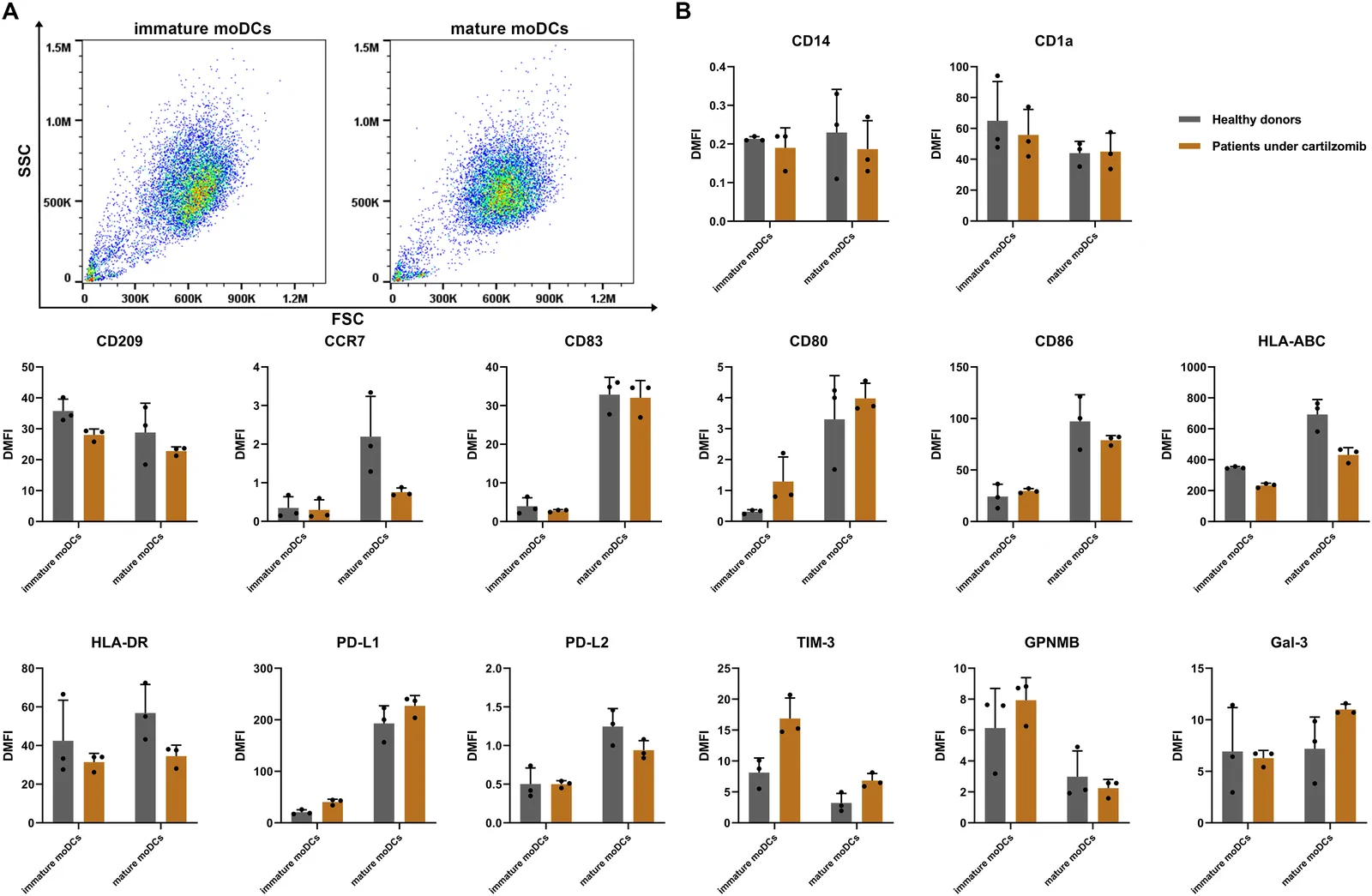
Generation of moDCs from carfilzomib-treated MM patients. **(A)** MoDCs were generated from patients’ monocytes undergoing carfilzomib/lenalidomide/dexamethasone treatment using the standard protocol with IL-4 and GM-CSF. Cell size and granularity were examined by FACS. Exemplarily, one representative for three patients is depicted. **(B)** Expression of the indicated cell surface markers characterizing moDC phenotype was investigated by FACS. Expression levels are shown as DMFI. Statistical analysis comparing three patients with three healthy controls is shown. (Mann-Whitney U test, Mean ± SD, no statistical significance).

### Assessment of DC subsets in MM patients treated with carfilzomib-based regimens

Moving forward in clinical translation, we analyzed a possible influence of carfilzomib on quantities of peripheral blood DC subsets in 44 patients, including 15 newly diagnosed MM patients, 11 patients receiving carfilzomib treatment, and 18 patients in remission. 22 healthy donors were analyzed for comparison.

DC subsets were identified by expression of CD1c and CD141 for cDC subsets. cDC2 were defined as CD45+, CD3-, CD19-, CD14-, and HLA-DR+, CD11c+, CD1c+, cDC1 as CD16-, CD141+ cells, and pDCs by high expression of CD123 and CD303 (**Supplementary Figure 3**). Total blood DC numbers were calculated as the sum of all subsets.

Frequencies of total blood DCs, cDC2, and pDCs, as well as absolute numbers of all three DC subtypes, were significantly reduced in newly diagnosed MM patients compared to healthy donors. With the exception of pDCs, DC frequencies during carfilzomib treatment and in patients in remission were comparable to healthy donors. Absolute numbers of total DCs, cDC2, and pDCs were slightly reduced during treatment and remission, whereas absolute cDC1 numbers were comparable to healthy donors (**Table 1**; **Figures 5A–C**).

**Table 1:** Blood DC numbers.

Group	In PBMCs (%)		In WBCs (%)		Absolute number (*10^7^/L)	
	Mean (range)	P-value	Mean (range)	P-value	Mean (range)	P-value
Total DC						
Healthy donors	1.04 (0.79-1.38)		0.40 (0.23-0.56)		2.88 (1.96-4.27)	
Newly diagnosed patients	0.50 (0.23-0.88)	< 0.0001	0.18 (0.07-0.35)	< 0.0001	1.03 (0.34-2.09)	< 0.0001
Patient under carfilzomib	0.94 (0.70-1.19)	0.4204	0.35 (0.21-0.58)	0.5625	1.42 (0.88-3.15)	< 0.0001
Patients in remission	0.88 (0.57-1.38)	0.0260	0.39 (0.22-0.65)	0.9996	1.36 (0.80-4.46)	< 0.0001
cDC1						
Healthy donor	0.040 (0.017-0.080)		0.016 (0.005-0.034)		0.118 (0.047-0.248)	
Newly diagnosed patients	0.015 (0.001-0.049)	0.0627	0.004 (0.000-0.011)	0.0541	0.032 (0.002-0.118)	0.0002
Patients under carfilzomib	0.068 (0.012-0.181)	0.0532	0.027 (0.009-0.068)	0.1015	0.110 (0.021-0.230)	0.9689
Patients in remission	0.047 (0.001-0.119)	0.8413	0.022 (0.000-0.063)	0.4456	0.083 (0.010-0.202)	0.1606
cDC2						
Healthy donors	0.60 (0.45-0.87)		0.23 (0.11-0.35)		1.670 (0.765-2.790)	
Newly diagnosed patients	0.34(0.13-0.56)	< 0.0001	0.13 (0.04-0.28)	0.0011	0.718 (0.258-1.401)	< 0.0001
Patients under carfilzomib	0.69 (0.52-0.84)	0.5264	0.25 (0.16-0.42)	0.7058	1.033 (0.764-2.033)	0.0004
Patients in remission	0.58 (0.35-0.93)	0.3045	0.26 (0.13-0.42)	0.4040	0.890 (0.564-2.972)	< 0.0001
pDCs						
Healthy donors	0.41 (0.21-0.77)		0.16 (0.06-0.31)		1.094 (0.588-2.015)	
Newly diagnosed patients	0.18 (0.03-0.35)	0.0009	0.05 (0.01-0.11)	< 0.0001	0.278 (0.068-0.698)	< 0.0001
Patients under carfilzomib	0.14 (0.02-0.34)	0.0001	0.07 (0.01-0.11)	0.0003	0.280 (0.026-0.918)	< 0.0001
Patients in remission	0.20 (0.01-0.44)	< 0.0001	0.11 (0.00-0.20)	0.0595	0.499 (0.011-1.403)	< 0.0001

**Figure 5:**
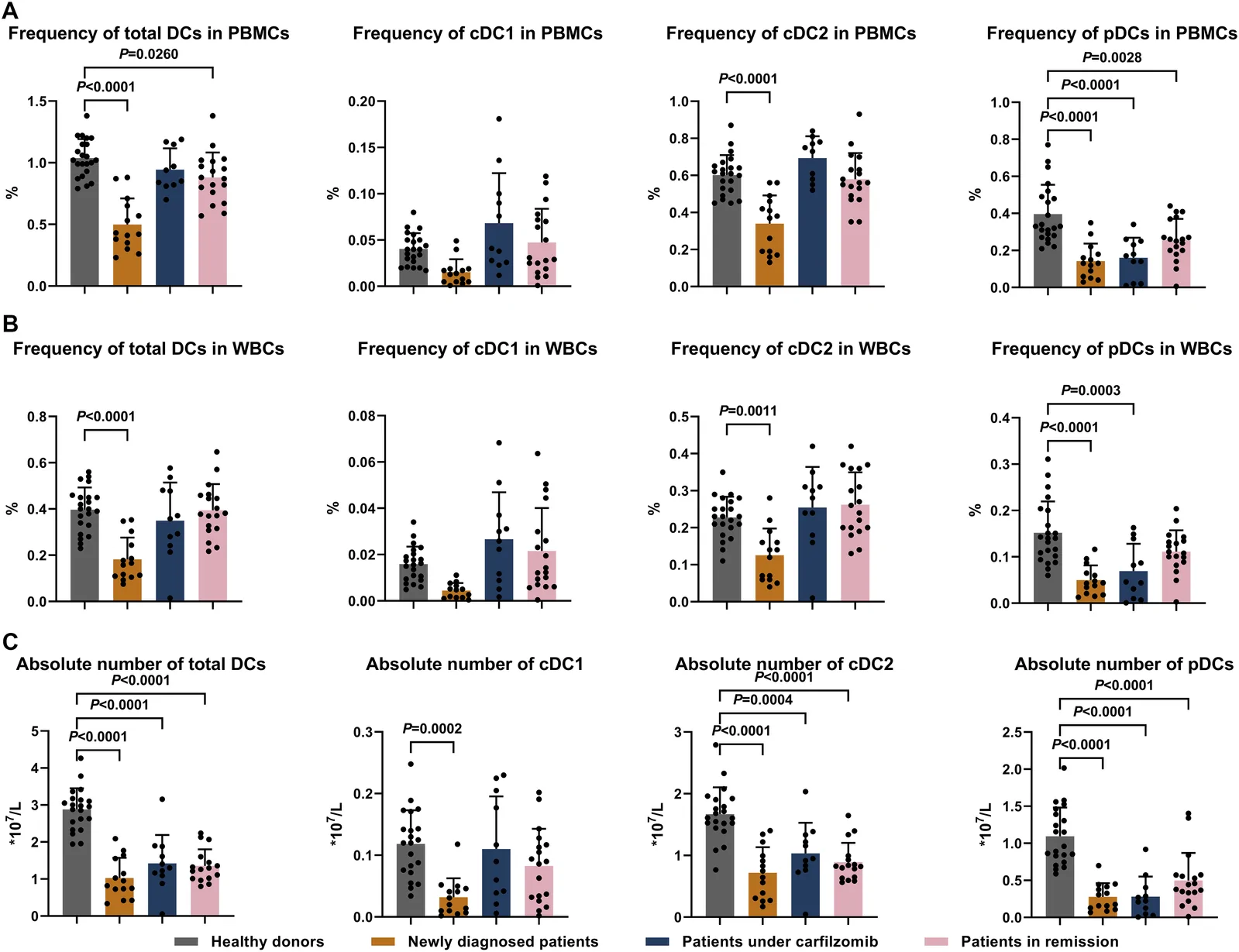
Quantification of blood DC subsets in healthy controls and patients by FACS. Comparison of blood DC numbers between healthy donors (n = 22), newly diagnosed MM patients (n = 15), patients under carfilzomib (n = 11), and patients in remission (n = 18). Data are shown as **(A)** frequency of DC subsets in PBMCs, **(B)** frequency of DC subsets in WBCs, and **(C)** absolute numbers of DC subsets. Mean percentages and cell numbers are shown in **Table 1**. (One-way ANOVA, Mean ± SD).

In newly diagnosed MM patients, lower total circulating DC numbers correlated with higher disease burden, as reflected by serum beta-2 microglobulin levels (p = 0.021) and the percentage of bone marrow plasma cell infiltration (p = 0.016). Clinical characteristics of the patient cohort are summarized in **Table 2**.

**Table 2:** Summary of newly diagnosed MM patient characteristics and correlation between total DC number in PBMC and MM parameters.

Clinical characteristics	Category	Total patients 15, N	P-value
Male		13	
Female		2	
Age, years median, range		65 (42–87)	0.071^a^
Heavy chain, n M-Ig (Immunoglobulin)	IgG	6	
	IgM	0	
	IgA	3	
	Light chain only	6	
Light chain, n	Kappa	6	
	Lambda	4	
	none	5	
Serum M-Ig median, range (g/L)		17.43 (0-70.00)	0.262^a^
Beta-2 microglobulin median, range (mg/L)		3.1 (1.8-41.6)	0.021^a^ r=-0.587
Plasma cells bone marrow infiltration median, range (%)		50 (4–90)	0.016^a^ r=-0.610
Calcium median, range (mmol/L)		2.30 (2.17-2.67)	0.514^a^
Creatinine median, range (mg/dL)		0.87 (0.52-6.68)	0.090^a^
LDH median, range (U/L)		241 (103–495)	0.825^a^
Hemoglobin median, range (g/dL)		10.2 (7.6-14.2)	0.408^a^
Osteolytic bone lesion, n	None	5	0.839^b^
	One or more	10	
Chromosome analysis, n	Standard risk	8	0.875^b^
	High risk	7	
R-ISS classification, n	1	3	0.565^c^
	2	8	
	3	4	

^a^ P-value for Spearman test. ^b^P-value for Mann-Whitney U test. ^c^P-value for one-way ANOVA.

Bone marrow DC frequencies were further analyzed in six newly diagnosed MM patients (diagnosed and treated at Charité - Universitaetsmedizin Berlin) and compared to healthy controls using published reference data (46, 47). Frequencies were calculated from total CD45+ bone marrow mononuclear cells excluding CD138+ myeloma cells. All DC subsets were reduced in MM bone marrow compared to healthy controls, including cDC1 (0.030% [0.016 - 0.040%] vs. 0.081%), cDC2 (0.43% [0.26 - 0.77%] vs. 0.60%), and pDCs (0.11% [0.06 - 0.37%] vs. 4.35%) (47) (**Table 3**). In summary, circulating DC numbers were reduced at MM diagnosis and especially the cDC1 and cDC2 subsets were comparable to patients under carfilzomib treatment and in remission (46).

**Table 3:** Bone marrow DC frequencies in BMMC of newly diagnosed MM patients (n=6).

Parameter	cDC1	cDC2	pDC
Median (range)%	0.030 (0.016-0.040)	0.43 (0.26-0.71)	0.11 (0.06-0.37)

## Discussion

MM remains a largely incurable malignancy despite the rapid expansion of therapeutic options over the past decade. While contemporary regimens can achieve deep initial responses, durable disease control is still rare and cumulative toxicity limits treatment options, particularly in elderly and frail patients. These challenges underscore the urgent need for therapeutic strategies that combine sustained efficacy with acceptable tolerability and restore effective antitumor immunity (48). In this context, DC-based cancer vaccines represent an attractive strategy, yet their clinical efficacy as monotherapy has been modest. Several studies have shown that this immunotherapeutic approach can prolong progression-free survival and, in some cases overall survival in various cancers when combined with chemotherapy, radiation therapy, or ICIs (49–51). The best results so far were achieved in an adjuvant setting (52, 53). Based on the biological rationale that DCs induce tumor-specific T cell responses and immune checkpoint blockade can prevent these T cells from being suppressed, further trials are currently testing the synergy of ICIs in combination with DC-based vaccines (NCT06751849, NCT02678741).

Carfilzomib is an approved second-generation PI and standard of care for first-line treatment in MM and could represent an ideal candidate for such combination approaches, as PIs have been demonstrated to induce so-called immunogenic cell death (19, 20, 54, 55). However, more data are needed to allow the choice of the most suitable PI and timing of vaccine application in combinatorial approaches.

For this, we systematically analyzed the phenotype and function of human moDCs exposed to carfilzomib and bortezomib.

The central and novel finding of this study is that exposure of moDCs to carfilzomib did not lead to the broad impairment of DC phenotype and function that we and others observe with bortezomib.

Previous studies have consistently shown that bortezomib exerts detrimental off-target effects in DCs, including impaired maturation, reduced migratory capacity, diminished cytokine production, and compromised T cell priming (23, 56, 57), thereby negatively affecting its use in combination with DC-based cancer vaccines, despite its advantageous ability to induce immunogenic cell death.

Our data confirm these findings regarding bortezomib-mediated effects. Using a comprehensive panel of phenotypic and functional assays, we detected no significant effect of carfilzomib on DC differentiation, maturation, antigen uptake, migration, or cytokine secretion.

We chose two different conditions for drug exposure in order to approximate the clinical pharmacokinetics of each drug. Carfilzomib is an irreversible epoxyketone administered as a short infusion and has a plasma half-life under 1 h, so we used a brief pulse followed by washout in our experimental setup to reproduce exposure of bystander immune cells (e.g. DCs) *in vivo*. Bortezomib, on the other hand, is a slowly reversible boronate with a long terminal half-life. Hence, we chose the 24 h condition as a pharmacologically appropriate setup. As shown in target-engagement experiments measuring β5 proteasome activity in mature and immature moDCs, the observed effects are not attributable to lower proteasome inhibition by carfilzomib under the treatment conditions compared to bortezomib. Furthermore, as carfilzomib was washed out after the 1 h exposure, and cells were collected 23 h later, we observed persistence of β5 inhibition beyond the period of drug exposure, consistent with the irreversible nature of carfilzomib binding.

Carfilzomib has a greater selectivity for the proteasome β5 subunit, whereas bortezomib additionally inhibits β1 and β2 activities. Here, we only measured β5 activity; we did not assess the contribution of β1 and β2 inhibition. We hypothesize that bortezomib’s broader proteasome inhibition may disproportionately affect antigen processing, cytoskeletal dynamics, and NF-κB-dependent signaling pathways that are essential for DC function. Although carfilzomib binds irreversibly to the proteasome, this pharmacological characteristic does not translate into increased DC toxicity or functional impairment, supporting clinical observations of improved tolerability compared to bortezomib (19).

Notably, carfilzomib exposure even led to increased CCR7 expression in immature moDCs and modest upregulation of the maturation marker CD83, reinforcing the notion that carfilzomib does not induce dysfunctional DC maturation but may lead to partial functional priming, potentially enhancing DC migratory capacity toward secondary lymphoid organs. Such priming could be advantageous in the context of vaccination strategies, where efficient DC trafficking is a prerequisite for effective T cell induction, as it has previously been shown that only a small proportion of ex vivo-generated DCs reach secondary lymphoid organs (58).

A key mechanistic insight from this study is the observation that carfilzomib induces PD-L1 upregulation in moDCs. While this was associated with a modest reduction in T cell stimulatory capacity in MLR, PD-1 blockade with nivolumab not only restored but significantly enhanced T cell proliferation *in vitro*, consistent with our data on GPNMB upregulation in TKI-treated DCs (43). In line with observed reduced proliferation, our data further show that IFN-γ production by T cells co-cultured with carfilzomib-exposed moDCs was significantly reduced (by 34%, P = 0.0240).

Within each donor, all 4 conditions used the same T cells, the same CD3/CD28 pre-activation was performed in the 2 antibody arms, and the same nivolumab concentration was used. The only variable differing is the DC used. Hence, any T cell intrinsic effect of nivolumab is present in both antibody arms and we claim the significantly greater effect of nivolumab on carfilzomib-treated moDCs can be attributed to the DCs themselves rather than T cells.

Our finding has several important implications. First, it suggests that carfilzomib-induced PD-L1 expression represents a dominant, but reversible, inhibitory mechanism rather than a global impairment of DC function. Second, it provides a possible mechanistic explanation for the limited efficacy of ICIs as monotherapy in MM (59). By contrast, a strategy combining carfilzomib-mediated immunogenic cell death, DC-based antigen presentation, and checkpoint blockade may synergistically overcome multiple layers of immune suppression.

ICIs used in combination with DC-based vaccines were shown to enhance vaccine efficacy in a multiple myeloma mouse model (60). Chu et al. reported in a preclinical study that the combination of DC vaccination, pomalidomide, dexamethasone, and a PD-L1 inhibitor achieved initial efficacy in MM (61). The observed downregulation of TIM-3 on immature DCs following carfilzomib exposure further supports a net shift toward enhanced DC immunogenicity. Given recent evidence that TIM-3 limits DC sensing of extracellular DNA and dampens cGAS-STING signaling (41), reduced TIM-3 expression may facilitate innate immune activation and antigen presentation, particularly in the context of immunogenic cell death.

To our knowledge, this is the first detailed comparative analysis of carfilzomib effects on human DC function.

These *in vitro* observations are supported by our preliminary translational data showing the feasibility of generating cells with a moDC-like phenotype from monocytes of carfilzomib-treated patients.

Our patient-derived data highlight an additional clinically relevant aspect: the numbers of circulating total DCs, cDC2, and pDCs are markedly reduced at MM diagnosis, correlating with disease burden. However, numbers of circulating DC subsets in patients receiving carfilzomib-based regimens and in remission patients were comparable to healthy donors.

This is of importance, as natural blood DCs possess superior circul-presentation capacity and are therefore increasingly favored over moDCs in recent vaccination trials (5, 62, 63), despite greater practicality of moDC generation in terms of timing, cost and scale. Consistent with this, comprehensive reviews report significant depletion of cDCs and pDCs in peripheral blood in MM, attributed in part to abundant immunosuppressive cytokines in the bone marrow microenvironment produced by myeloma cells (47, 64).

This observation might have direct implications for vaccine timing. DC-based immunotherapy may be most effective after initial reduction of tumor burden, when immune competence is partially restored, tumor-mediated suppression is reduced, and DC populations recover (65). Carfilzomib-based induction therapy, therefore, may create a permissive immunological window for vaccinations. This hypothesis requires further validation but is supported by clinical studies demonstrating improved outcomes when DC vaccines are administered after induction therapy or autologous stem cell transplantation (17, 66, 67). Of note, the analyses we performed are cross-sectional associations of blood and bone marrow DC frequencies with disease and treatment status and hence cannot exclude effects of the other drugs contained in the treatment regimens. To our knowledge, these are novel data on blood DC frequencies of MM patients receiving carfilzomib-based regimens.

Importantly, we demonstrate that moDCs can be generated from patients receiving carfilzomib-containing regimens without loss of viability or phenotypic integrity, supporting the feasibility of autologous DC vaccines in this setting. While patients in remission continued to exhibit lower absolute DC numbers than healthy controls, this likely reflects prior myelotoxic therapies and may not preclude effective vaccination (**Supplementary Table 2**). Notably, Shinde et al. documented compromised moDC yields, migration, and cytokine secretion in pre-transplant patients (68). This discrepancy may be attributed to the intensive chemotherapy regimens administered before transplantation in their patients, which are known to be broadly myelosuppressive and detrimental to immune cell populations.

Hence, our data support the broader use of DC-based cancer vaccines, even at an early point during carfilzomib treatment, thereby not restricting potential benefit to patients who have already responded well to available therapies.

DC-based vaccines are increasingly revisited in the era of immune checkpoint inhibition with growing recognition that effective antigen presentation is a prerequisite for durable immune responses. Our findings align with emerging clinical and preclinical studies showing that DC vaccines can sensitize tumors to PD-1/PD-L1 blockade (69, 70). In MM, where checkpoint inhibitors alone have shown limited efficacy, rational combination strategies are urgently needed. One hypothesis for further studies could be that carfilzomib represents as an immunologically compatible backbone therapy that both induces ICD and largely preserves DC competence, thereby enabling effective integration of DC vaccines and checkpoint blockade.

Furthermore, MM patients are commonly treated with a triple or quadruple combination including immunomodulatory drugs (IMiDs) as in our studies. Several trials showed that IMiDs enhance immune responses (71). However, adding checkpoint inhibition to these strategies urgently requires further investigation, particularly regarding optimal timing. This is of special importance as the FDA halted KEYNOTE-185 and -183 clinical trials testing combination approaches of IMiDs and PD-1 inhibition due to safety issues potentially related to immune overstimulation (72, 73).

Besides the restriction already mentioned, our study has several limitations that should be acknowledged. Although our clinical analysis included the enumeration of naturally circulating DC subsets, the mechanistic *in vitro* experiments were performed using moDCs, which may not fully recapitulate the phenotype and function of naturally circulating DC subsets, whose function we did not analyze. In addition, the number of patient samples analyzed for ex vivo generation of moDCs (n = 3) and DC subsets (newly diagnosed MM patients n = 15, patients under carfilzomib n = 11, and patients in remission n = 18, bone marrow newly diagnosed MM patients n = 6) was relatively small, and further investigation is needed to analyze functional characteristics of naturally circulating and bone marrow DC subsets, as well as moDCs derived from patient PBMCs also in terms of antigen-specific priming in larger patient cohorts, which was beyond the scope of this study. Further work should also focus on *in vivo* validation of DC-checkpoint inhibitor synergy and on prospective clinical trials testing DC-based vaccines, including personalized neoantigen strategies combined with carfilzomib-based regimens and PD-1 blockade, in early-stage or minimal disease settings.

In summary, our study suggests that carfilzomib does not reproduce the broad impairment caused by bortezomib on DC phenotype and function. The discrete deficit it does produce is fully reversed and significantly exceeded by PD-1 blockade. We further provide insight into blood DC frequencies and observed that DC numbers were not reduced in patients receiving carfilzomib-based regimens, which is potentially important for the timing of vaccination. Our first preliminary results show that the generation of cells with moDC phenotype using monocytes of these patients is possible.

We hypothesize that carfilzomib represents a more suitable proteasome inhibitor partner than bortezomib for DC-based vaccination strategies, and that combination with checkpoint-blockade may enhance their clinical efficacy.

## Author’s note

Data of this paper had in part been presented at the Annual Meeting of the German, Austrian and Swiss Societies for Hematology and Oncology, Basel, Switzerland, 2015 Einfluß des Proteasominhibitors Carfilzomib auf humane dendritische Zellen. Dahlfrancis, Marius, Dörfel D., Stickel J.S., Walz S., Grünebach F., Rittig S.M. P812 (74).

## Data Availability

The original contributions presented in the study are included in the article/**Supplementary Material**. Further inquiries can be directed to the corresponding author.
