# Supplementary material for: Carfilzomib preserves dendritic cell phenotype and function compared with bortezomib
Source: Front Immunol. 2026 Sep 22;17:1936021. doi: 10.3389/fimmu.2026.1936021 (PMC13638640; doi:10.3389/fimmu.2026.1936021)
Supplement: Supplementary file 1 [file SupplementaryFile1.docx]

Supplementary Material

**Supplementary Figure 1.** Cell viability under carfilzomib exposure. (A) The U266 multiple myeloma cell line was treated with the indicated concentrations of carfilzomib. Thereafter, cells were harvested and analyzed by FACS using Annexin-V and propidium iodide staining. (B) Mature and immature moDCs were exposed to 100nM of carfilzomib for 1 hour or DMSO control. Cells were then stained with Annexin-V and propidium iodide and analyzed by FACS. (Mann-Whitney U test, Mean ± SD, no statistical significance) N=3. (C) Exemplary results of the effect of 100 nM carfilzomib on U266 and moDC cell viability are shown by FACS dot plots using an Annexin/propidium iodide assay.

**Supplementary Figure 2.** Proteasome activity under carfilzomib and bortezomib treatment. Proteasome activity of β5 subunit was measured in immature and mature moDC lysates following treatment with carfilzomib (100 nM, 1 h) or bortezomib (1 ng/ml, 24 h). β5 Activity was analyzed using Proteasome-Glo assay and normalized to the DMSO control. (Two-way ANOVA, Mean ± SD, n=3).

**Supplementary Figure 3.** Gating strategy for DC subsets. CD45+ mononuclear cells (MNCs) were gated after doublet exclusion. Thereafter, cell subsets were identified based on the expression of specific surface markers CD16-, CD141+ (cDC1); CD11c+, CD1c+ (cDC2); CD123+, CD303+ (pDCs).

**Supplementary Table 1.** Treatment characteristics of individual patients under carfilzomib

| Patient code | Treatment regimen | Treatment cycle | Lines of therapy |
| --- | --- | --- | --- |
| GC | Kd | 6 | First line |
| HS | KRd | 4 | First line |
| PC | KRd | 6 | First line |
| HPL | KRd | 7 | First line |
| KDS | KRd + Isatuximab | 5 | First line |
| BZ | KRd | 5 | First line |
| MP | KRd + Isatuximab | 6 | First line |
| MH | KRd + Cyclophosphamide | 5 | First line |
| GP | KRd | 5 | First line |
| MEH | KRd | 4 | First line |
| NP | KRd | 5 | First line |

**Supplementary Table 2.** Number of prior lines of therapy of patients in remission

| Patient code | Prior lines of therapy |
| --- | --- |
| EB | 4 |
| MG | 8 |
| PH | 3 |
| AB | 4 |
| CB | 4 |
| JF | 3 |
| RT | 3 |
| RK | 4 |
| WS | 5 |
| VH | 3 |
| BE | 2 |
| FL | 3 |
| AS | 3 |
| GH | 4 |
| SK | 5 |
| MP-R | 3 |
| LB | 3 |
| SO | 2 |
